# Phosphatidylserine and Phosphatidylethanolamine Bind to Protein Z Cooperatively and with Equal Affinity

**DOI:** 10.1371/journal.pone.0161896

**Published:** 2016-09-01

**Authors:** Tanusree Sengupta, Narayanan Manoj

**Affiliations:** Department of Biotechnology, Bhupat and Jyoti Mehta School of Biosciences, Indian Institute of Technology Madras, Chennai—600036, India; Griffith University, AUSTRALIA

## Abstract

Protein Z (PZ) is an anticoagulant that binds with high affinity to Protein Z-dependent protease inhibitor (ZPI) and accelerates the rate of ZPI-mediated inhibition of factor Xa (fXa) by more than 1000-fold in the presence of Ca^2+^ and phospholipids. PZ promotion of the ZPI-fXa interaction results from the anchoring of the Gla domain of PZ onto phospholipid surfaces and positioning the bound ZPI in close proximity to the Gla-anchored fXa, forming a ternary complex of PZ/ZPI/fXa. Although interaction of PZ with phospholipid membrane appears to be absolutely crucial for its cofactor activity, little is known about the binding of different phospholipids to PZ. The present study was conceived to understand the interaction of different phospholipids with PZ. Experiments with both soluble lipids and model membranes revealed that PZ binds to phosphatidylserine (PS) and phosphatidylethanolamine (PE) with equal affinity (*K*_d_~48 μM); further, PS and PE bound to PZ synergistically. Equilibrium dialysis experiments revealed two lipid-binding sites for both PS and PE. PZ binds with weaker affinity to other phospholipids, *e*.*g*., phosphatidic acid, phosphatidylglycerol, phosphatidylcholine and binding of these lipids is not synergistic with respect to PS. Both PS and PE -containing membranes supported the formation of a fXa-PZ complex. PZ protection of fXa from antithrombin inhibition were also shown to be comparable in presence of both PS: PC and PE: PC membranes. These findings are particularly important and intriguing since they suggest a special affinity of PZ, *in vivo*, towards activated platelets, the primary membrane involved in blood coagulation process.

## Introduction

Human Protein Z (PZ) is a multi-domain, 62 kDa, vitamin K-dependent cofactor that promotes ZPI (protein Z dependent Protease Inhibitor)-mediated inhibition of factor Xa (fXa), the central enzyme of the blood coagulation cascade [1]. Although human PZ was first isolated in 1984, its precise physiological role is still unclear [2]. PZ deficiency dramatically increases the severity of the prothrombotic phenotype of factor V Leiden mice [3]. Apart from its catalytic role in promoting the membrane-associated ZPI-fXa reaction, PZ in complex with ZPI may inhibit tissue deposition of fibrin and act as an anti-inflammatory agent [4, 5]. The N-terminal half of PZ containing a γ-carboxy glutamic acid-rich (Gla) domain and two epidermal growth factor-like domains (EGF1 and EGF2) is homologous to factors VIIa, IXa, and Xa. However, His and Ser residues of the catalytic triad present in other coagulation proteases are not conserved in the homologous region in PZ [6]. Thus, although PZ has no catalytic function, it accelerates ZPI- mediated inhibition of fXa by ~1000 fold in the presence of pro-coagulant lipid and Ca^2+^. Presence of Ca^2+^ is essential as it orients PZ and ZPI in an appropriate position with respect to the membrane [7]. Rezaie et al. noticed a remarkable reduction in the cofactor activity of Gla domain-less PZ (GD-PZ) on PC (phosphatidylcholine) /PS (phosphatidylserine) vesicles, although the mutant exhibited a normal cofactor activity in solution [8]. These investigators also demonstrated that, in the presence of Ca^2+^and PC/PS vesicles, an activated protein C (APC) chimera containing the Gla domain of fXa binds with ZPI in the presence, but not in the absence of PZ. Wei et al. have also shown that GD-PZ is incapable of accelerating the ZPI-mediated inhibition of fXa [9]. Therefore, it is evident that PZ binds firmly to the phospholipid membrane and forms a stabilizing interaction with fXa bound to the same membrane [10]. The cofactor activity of PZ thus results from the anchoring of its Gla domain onto the phospholipid surface and juxtaposing ZPI close to fXa. Interaction of fXa and PZ on the phospholipid surface also leads to the slowdown of the rate of inhibition of fXa by antithrombin (AT), another well known serpin [1].

Phospholipid membranes are known to play critical roles in the assembly and formation of blood clotting protease-cofactor complexes such as prothrombinase (fXa-fVa) and intrinsic fXase (fIXa-fVIIIa). The platelet membrane is by far the primary membrane affecting blood coagulation, however, sub-endothelial cell membranes and endothelial cell membranes also contribute towards the initiation and shut down of coagulation. Based on clotting assays, synthetic phospholipid vesicles containing either PS or PA (phosphatidic acid) can replace platelet membrane in platelet-free plasma [11]. While PS is the most significant phospholipid that regulates the structure and function of coagulation proteins such as fXa, fIXa, and fVa [12–14], other phospholipids also play crucial roles in coagulation. PE, another phospholipid abundant in activated platelet, was reported to modulate APC-mediated inactivation of fVa [15], promote the assembly and activity of the prothrombinase (fXa-fVa) complex [16], and regulate the structure and activity of fVIIa [17], (unpublished data). Again, fVIIa and activated protein C (APC) were reported to preferentially bind to nanobilayers containing PA [18]. Phosphatidylinositol 4-phosphate (PIP) also supported enhanced enzymatic activity of fVIIa and APC. Thus, there exist systematic studies of the interaction of lipid membranes with most coagulation proteins. However, little data are available that describe the interaction and binding of PZ to membrane phospholipids [19]. Most studies with the PZ/ZPI system have focused on the interaction between PZ and ZPI and between ZPI and fXa [20–22].

Because phospholipids are critical for the function of the PZ-ZPI-fXa system, we have measured the PZ binding properties of different phospholipids with primary focus on PS and PE. Binding experiments employed both small unilamellar vesicles (SUV) of varying lipid composition and six-carbon chain soluble forms of PS (C6PS, 1, 2-dicaproyl-sn-glycero-3-phospho-L-serine), PE (C6PE, 1, 2-dicaproyl-sn-glycero-3-phospho-L-ethanolamine) and other lipids. We observed PS and PE to bind at different and distinct sites on PZ with similar affinities in a Ca^2+^ dependent manner; stoichiometry of binding being 2 for both the lipids. Furthermore, these lipids synergistically enhance the binding of each other. Binding affinities of other soluble lipids (C6PC, C6PG, C6PA) to PZ is lower by ~3-fold compared to PS and PE. PZ had similar protective effects on fXa inhibition by AT (Antithrombin) in presence of both PS and PE. Finally, either PS or PE containing membranes are absolutely essential for the assembly and formation of fXa-PZ complex.

## Materials and Methods

### Materials

Human PZ was purchased from Enzyme Research Laboratories (South Bend, IN, USA). DEGR-Xa [(5-(Dimethylamino)-1-naphthalenesulfonyl]-Glutamylycylarginyl)-Xa] was from Haematologic Technologies Inc. (Essex Junction, VT, USA).1,2-dioleolyl-3-sn-phosphatidylcholine (DOPC), 1,2-dioleoly-3-sn-phosphatidylethanolamine (DOPE), 1,2-dioleoyl-3-sn-phosphatidylserine (DOPS), the sodium salts of 1, 2-dicaproyl-sn-glycero-3-phospho-L-serine (C6PS) and 1, 2-dicaproyl-sn-glycero-3-phospho-L-phosphatidyl ethanolamine (C6PE), and all other lipids were purchased from Avanti Polar Lipids (Alabaster, AL, USA). The fXa-specific substrate N-2-benzyloxycarbonyl-D-arginyl-L-arginine p-nitroanilide dihydrochloride (S-2765) was purchased from Diapharma (West Chester, OH, USA). All other chemicals were ACS reagent grade or the best available grade; all solvents were HPLC grade.

### Preparation of lipid samples

Small unilamellar vesicles (SUV) were prepared following the method of Bandari et al. with slight modifications [23]. Briefly, different phospholipids (DOPC, DOPE, DOPS) in chloroform were mixed at the desired molar ratio and dried under a stream of nitrogen. The resultant lipid films were dried for a further 2–3 h in a vacuum desiccator to remove remaining solvent. The dried films were hydrated in 20 mM Tris-HCl, pH 7.5, 150 mM NaCl for at least an hour with occasional vortexing. To ensure solute equilibration between trapped and bulk solutions, the hydrated mixtures were then freeze-thawed five times using liquid nitrogen and a 42°C water bath. Finally, the mixtures were extruded through an Avanti Extruder with a polycarbonate filter of 100 nm pore size and then through a filter of 30 nm pore size. Samples were subjected to 20 passes through each filter to generate the final SUV. Phospholipid concentrations of the SUV were determined according to the inorganic phosphate assay method [24].The sizes of the phospholipid vesicles were determined using a particle size analyzer instrument, Zetatrac, Microtrac, (USA). Phospholipid vesicles prepared using this method had an average diameter of 30–40 nm.

Soluble lipid suspensions of desired concentrations were prepared as described previously [25].

### Fluorescence measurements

PZ intrinsic tryptophan fluorescence as a function of increasing concentration of soluble phospholipid was measured as described in [12] using a FluoroLog spectrofluorometer (Horiba Jobin-Yvon Inc., Edison, NJ) with an excitation wavelength of 285nm (band-pass 4 nm); emission was recorded at 340 nm (band-pass 4 nm). Each experiment was performed below the critical micelle concentration (CMC) of the soluble lipid. The CMC was determined under experimental conditions following pyrene fluorescence as a function of increasing lipid concentration [26]. Apparent dissociation constants for binding of PZ to soluble lipids were obtained by fitting the experimental data to a simple, single-site binding model, using the assumption that [C6PL]_free_ ≈ [C6PL]_total_

DEGR-Xa fluorescence was recorded in presence of 50 μM lipid membrane and increasing concentration of PZ, using an excitation wavelength of 340 nm and emission wavelength of 545 nm (band pass 4 nm) [12].

### Equilibrium dialysis

The stoichiometry of soluble C6PS and C6PE binding to PZ in the presence of 5 mM Ca^2+^ was determined by equilibrium dialysis measurements as described in [27,28]. Experiments were performed using 2.0-ml Teflon dialysis cells (Harvard Apparatus, Holliston, MA) with the two cells separated by a 2000 Da molecular weight cut-off membrane. Both chambers contained equal amount of C6PS/C6PE, and one chamber contained the appropriate amount of PZ. Both chambers were allowed to equilibrate with each other at room temperature for 24 h while being rotated horizontally at 20 rpm. The differences in phosphate concentration (ΔP) between the protein-free and protein-containing chambers were then measured and correlated as,

$$\Delta P=\frac{\left[\text{L}]\text{n}[\text{P}\right]}{K_{d}(1+[\text{L}]/K_{d})}$$ (1)

where, n is the stoichiometry of binding, [L] is the concentration of phospholipid added to the chamber, [P] is the protein concentration present in one half of the chamber, and *K*_d_ is the binding constant obtained from fluorescence experiments. Total protein concentration, [P]_,_ must be large enough to detect a measurable difference in phosphate concentration between the two chambers. In a recent analysis of soluble lipid binding to two non-equivalent sites on fIXa, it was shown that a narrow protein concentration range was sufficient to obtain stoichiometries for each site [14]. Here three different PZ concentrations (15, 20, 30 μM) were used with 250 μM C6PS/C6PE to obtain greater precision in determination of stoichiometry. For each protein concentration, three aliquots were withdrawn and phosphate concentrations were measured.

### Membrane binding of PZ

The binding of PZ to phospholipid vesicles was monitored using membranes labeled with 2.5 mol % dansyl-phosphatidylethanolamine (dansyl-PE) as described in [29, 30]. Fluorescence energy transfer from tryptophan of PZ to the membrane-located dansyl group was measured at excitation and emission wavelengths of 280 nm and 545nm, respectively.

### FXa inhibition assay

Amidolytic activity of human fXa was measured in an assay mixtures containing 5 nM fXa, 3 μM AT, 50 μM phospholipid membrane (DOPS:DOPC, DOPE:DOPC, and only DOPC) and 40 nM PZ in buffer (50 mM Tris, 175 mM NaCl, 0.6% PEG, 5 mM CaCl_2_, pH 7.4 using the substrate S2765 according to the method described in [31]. Briefly, mixtures (150 μl) containing fXa in appropriate buffer were incubated with or without PZ and with or without membrane at 37°C in the wells of a microtiter plate for 5 mins before adding AT to that. 1 mM of substrate S2765 was added then and initial rate of substrate cleavage was determined by measuring the absorbance at 405 nm using the plate reader (Molecular Devices, CA, USA). Factor Xa activity was determined by comparing the initial rate of substrate cleavage with a standard curve produced with various concentrations of factor Xa in the same buffer conditions.

## Results

### Binding of PZ to C6PS and C6PE

To assess the interaction of PZ with soluble phospholipids (C6PL), we measured the effect of individual phospholipids on PZ intrinsic tryptophan fluorescence. Although C6PS and C6PE do not occur physiologically, they have proven to be powerful tools to demonstrate the effect of binding molecular PS and PE to coagulation proteins. C6PS and PE binding offers invaluable insights into events that would be next to impossible to document on membranes. These lipids are successfully used to locate and characterize the lipid binding sites on fXa, fIXa and fVa [14, 16, 32]. In most cases, effect of molecular PS/PE on coagulation proteins are similar to that observed with membrane-associated proteins [25]. Therefore, it is quite reasonable to use those lipids for studying the interaction of different phospholipids with PZ.

**Fig 1:**
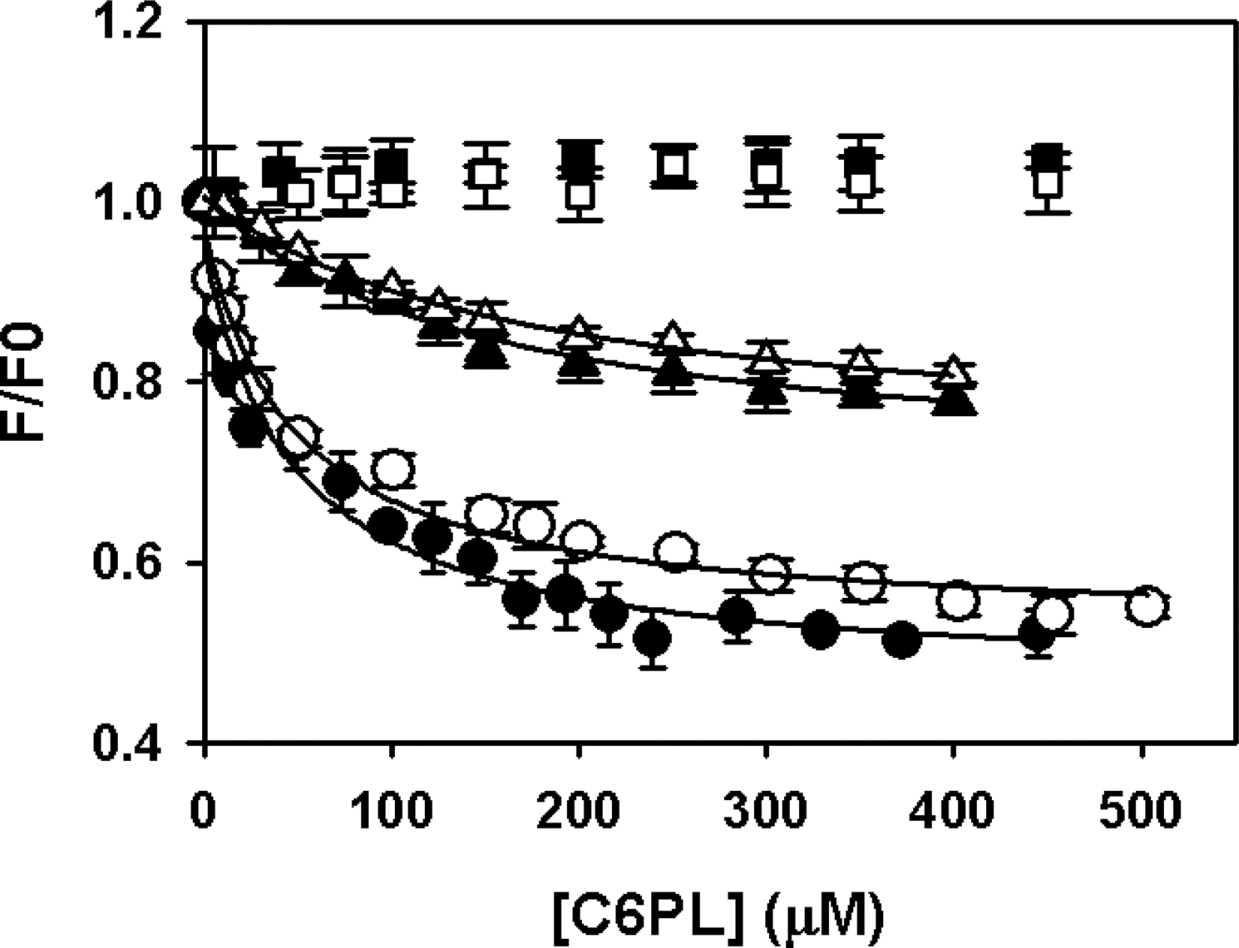
Binding of C6PS and C6PE to PZ measured by intrinsic fluorescence. To detect binding, intrinsic tryptophan fluorescence intensities of 150 nM PZ in 50 mM Tris-HCl, pH 7.5, 175 mM NaCl, 5 mM CaCl_2_, 0.6% PEG were measured at 25° C as a function of added C6PS (●) or C6PE (○). Solid lines show fits of the data to a simple single-site binding model, which predicted apparent *K*_d_ values for binding of C6PS and C6PE as 47.7 ± 10.7 μM and 47.6 ±8.2 μM, respectively. Fluorescence titrations were also performed under two more conditions: in presence of 5 mM EDTA with C6PS (■) and C6PE (□); and in presence of 1 mM Ca^2+^ with C6PS (▲) and C6PE (Δ).

The intensity of PZ fluorescence signal decreased with the addition of increasing amounts of C6PS and C6PE (Fig 1). The binding curves in both cases were described reasonably well by a single-site binding model with effective dissociation constants of 47.7 ± 10.7μM for C6PS and 47.6 ± 8.2 μM for C6PE. Fig 1 also shows that the net fluorescence change at saturation was similar for PS and PE indicating similar response and affinities for both lipids at saturation. We did not observe any binding when excess EDTA (5 mM) was added in the assay buffer confirming the interaction of phospholipids with PZ to be Ca^2+^ dependent (Fig 1). In presence of 1mM Ca^2+^, binding *K*_d_'s were measured to be 165 μM and 172 μM for C6PS and PE respectively, which are about 3.5 fold more than that obtained in presence of 5 mM Ca^2+^. Thus binding was again shown to be Ca^2+^dependent.

We used pyrene fluorescence to show that the lipid concentrations in the above experiments were all well below their critical micelle concentrations (CMC), thus confirming the absence of micelles. Since pyrene is hydrophobic, partitioning into the interior of a micelle is manifested as a sharp increase in the ratio of the intensity of the pyrene emissions at 373 and 383 nm. Under our experimental conditions, the CMCs of C6PS and C6PE were 950 μM and 1.2 mM, respectively, well above the conditions used in our study (data not shown).

**Fig 2:**
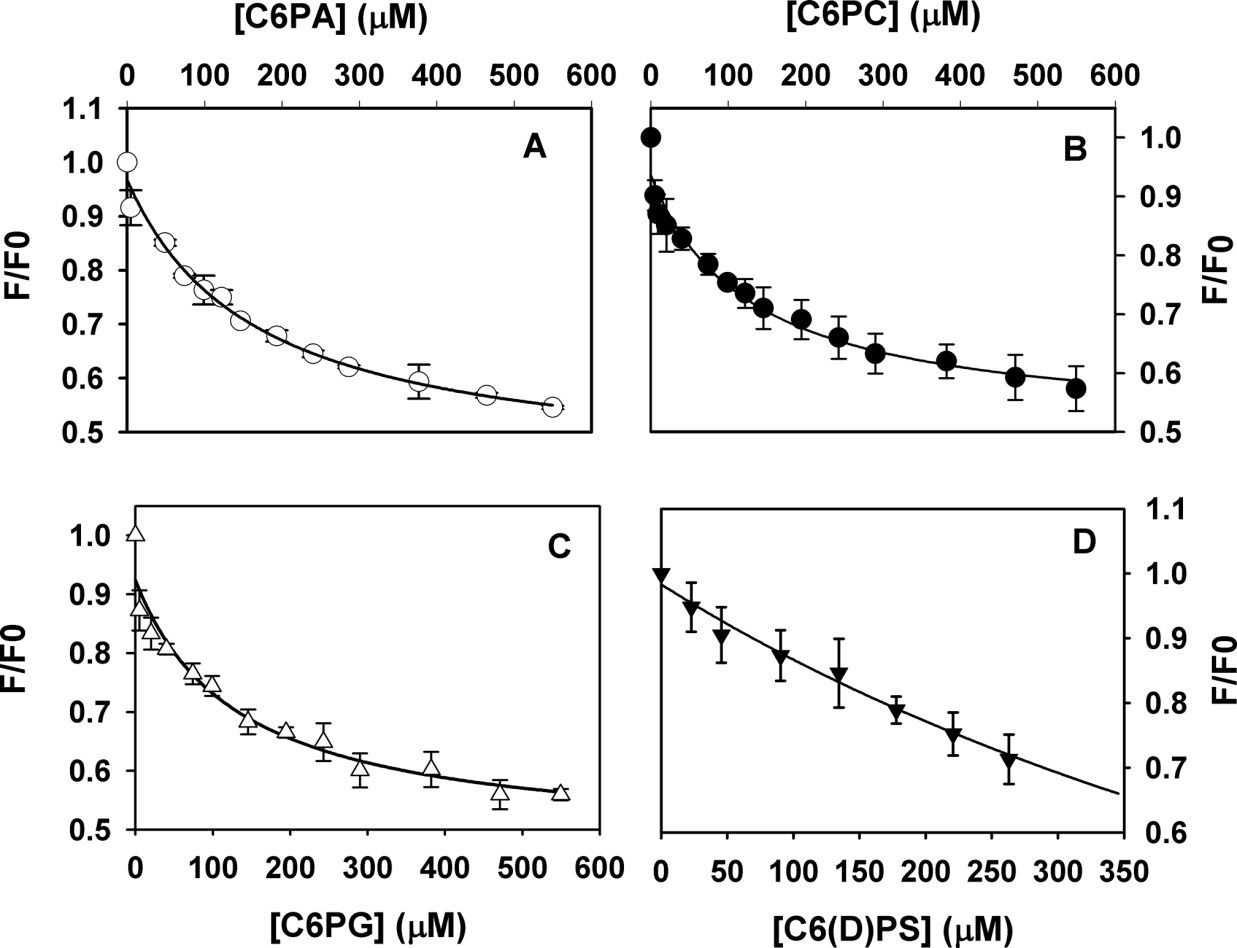
Binding of different six-carbon chain soluble lipids to PZ. Intrinsic tryptophan fluorescence intensities of 150 nM PZ in 50 mM Tris-HCl, pH 7.5, 175 mM NaCl, 5 mM CaCl_2_, 0.6% PEG were measured as a function of added A. C6PA (○), B. C6PC (●), C. C6PG (Δ) and D. C6(D)PS (▼) to obtain binding constants. The apparent *K*_d_ values for binding are 165 ± 25 μM, 129 ± 32, 131± 39, for PA, PC and PG respectively. (D)-PS shows extremely weak binding with *K*_d_~900 μM.

Binding to PZ was also observed for other soluble lipids such as C6PC, C6PA, C6PG and C6(D)PS (Fig 2). Fitting the data to the same single-site binding model yielded *K*_d_ values of 165 ± 25 μM, 129 ± 32 μM, 131± 39 μM for PA, PC and PG, respectively. It is evident from the binding *K*_d_ values that PZ has 2–3 fold lower affinity towards other soluble lipids compared to PS and PE. Binding of PZ to C6(D)PS, containing D-serine exhibited a *K*_d_ of 900 μM indicating that PZ, like most other coagulation proteins, preferentially recognizes PS containing L-serine.

### Stoichiometry of soluble phospholipid binding to PZ

Next we investigated the number of PS and PE binding sites present on PZ. The stoichiometries for binding of C6PS and C6PE to PZ were determined by equilibrium dialysis experiment as described in the Methods. The equilibrium dialysis data are formally expressed in terms of the difference in phosphate concentration (ΔP) between the protein-containing and protein-free chambers of the dialysis apparatus. Values calculated from the data using Eq (1) stated in the Methods are tabulated in Table 1. Our data suggests the existence of two PS binding sites and two PE binding sites on PZ. We did not detect any binding of PS or PE in the absence of Ca^2+^ and presence of EDTA in the buffer.

**Table 1:** Summary of the results obtained from equilibrium dialysis experiment.*

Lipid	[PZ] (μM)	ΔP	Stoichiometry (Average)
250 μM C6PS	15	27.6 ± 0.2	2.0 (1.96 ± 0.1)
	20	32.3 ± 0.09	
	30	45.3 ± 0.1	
250 μM C6PE	15	28.9 ± 0.08	2.0 (1.93 ± 0.2)
	20	31.8 ± 0.2	
	30	42.8 ± 0.6	

* Stoichiometry of lipid binding was measured using 250 μM C6PS/C6PE and three different protein concentrations. Three measurements were carried out for each protein concentration and average values of stoichiometry with standard errors calculated from Eq (1) are shown in the table.

### Synergistic binding of PS and PE to PZ

Previous reports have shown PE to synergize with PS in promoting the activity of extrinsic Xase complex (fVIIa-TF). PE is also effective in APC-mediated inactivation of fVa in the presence of a small amount of PS [15]. Further, Tavoosi et al. showed that phospholipids with any head-group other than choline strongly synergize with PS to escalate fX activation [33]. To understand if there is a synergy between PE and PS to interact with PZ, we incubated PZ with saturating concentration of C6PE and then titrated with C6PS. Fitting and analysis of the data yielded *K*_d_ value of 0.8 ± 0.02 μM for C6PS binding in the presence of C6PE which was much less compared to that obtained with C6PS alone. Therefore, binding of PE to PZ increases the binding affinity of PS towards PZ. Similar cooperativity of binding was also observed when PZ was titrated with C6PE after incubating with saturating amount of C6PS. Binding constant of C6PE to PZ, incubated with C6PS, was found to be 1.2 ± 0.15 μM, which is also much less compared to the binding *K*_d_ of C6PE alone. PE and PS were thus found to act synergistically to bind PZ. The data also implies that binding sites of PS and PE on PZ are different and distinct and may or may not be linked.

To determine whether this cooperative effect is specific for PS and PE, we performed the same experiment with C6PC, C6PG and C6PA in the presence of a saturating amount of C6PS. However, our data clearly indicated that pre-incubation of PZ with C6PS did not alter the affinity of PZ towards the other lipids, confirming that the synergistic enhancement is specific to PS and PE (Table 2).

**Table 2:** Linkage between sites for soluble lipids on human PZ.*

Lipid Titrant	Pre-incubation Lipid	*K*_d_(μM)
C6PS	----	47.7 ± 10.7
C6PE	----	47.6 ±8.2
C6PS	100 μM C6PE	0.8 ± 0.02
C6PE	100 μM C6PS	1.2 ±0.15
C6PA	----	165 ± 25
C6PA	100 μM C6PS	180 ±17
C6PC	----	129±32
C6PC	100 μM C6PS	132±21
C6PG	----	131±39
C6PG	100 μM C6PS	140 ±12
C6(D)PS	----	911 ± 300

*Binding of soluble phospholipids to 150 nM PZ was monitored using the intrinsic fluorescence intensity in 50mMTris-HCl, pH 7.5, 175 mM NaCl, 5 mM CaCl_2_ as a function of C6PS, C6PE, C6PA, C6PC, C6PG or C6(D)PS. The data were analyzed according to a single-site binding model to reveal the synergy in binding of these lipids.

### Binding of PZ to phospholipid membranes

Our soluble lipid binding data showed that PS and PE bind to PZ synergistically and with equal affinity. Binding of these two lipids were also observed to be stronger compared to other phospholipids. To substantiate our soluble lipid binding data, we monitored PZ binding to phospholipid vesicles composed of lipids with varying head group. We employed established FRET experiments to observe the binding of PZ to three different types of membranes (PS/PC, PE/PC, PS/PE/PC) of varying compositions, labeled with dansyl-PE. Control experiments were performed to account for any possible effect the probe might have on PZ binding to PS/PC membranes. Binding of PZ with dansyl-PE-labeled membranes of different phospholipid composition was monitored in the presence and absence of identical phospholipid membranes without the probe. Two data sets for each membrane composition were then compared after normalizing the data for the absolute amount of dansyl-PE in the membranes. For each membrane composition, both data sets followed the same trace, confirming that dansyl-PE does not have any effect on PZ binding to PS/PC membranes.

**Fig 3:**
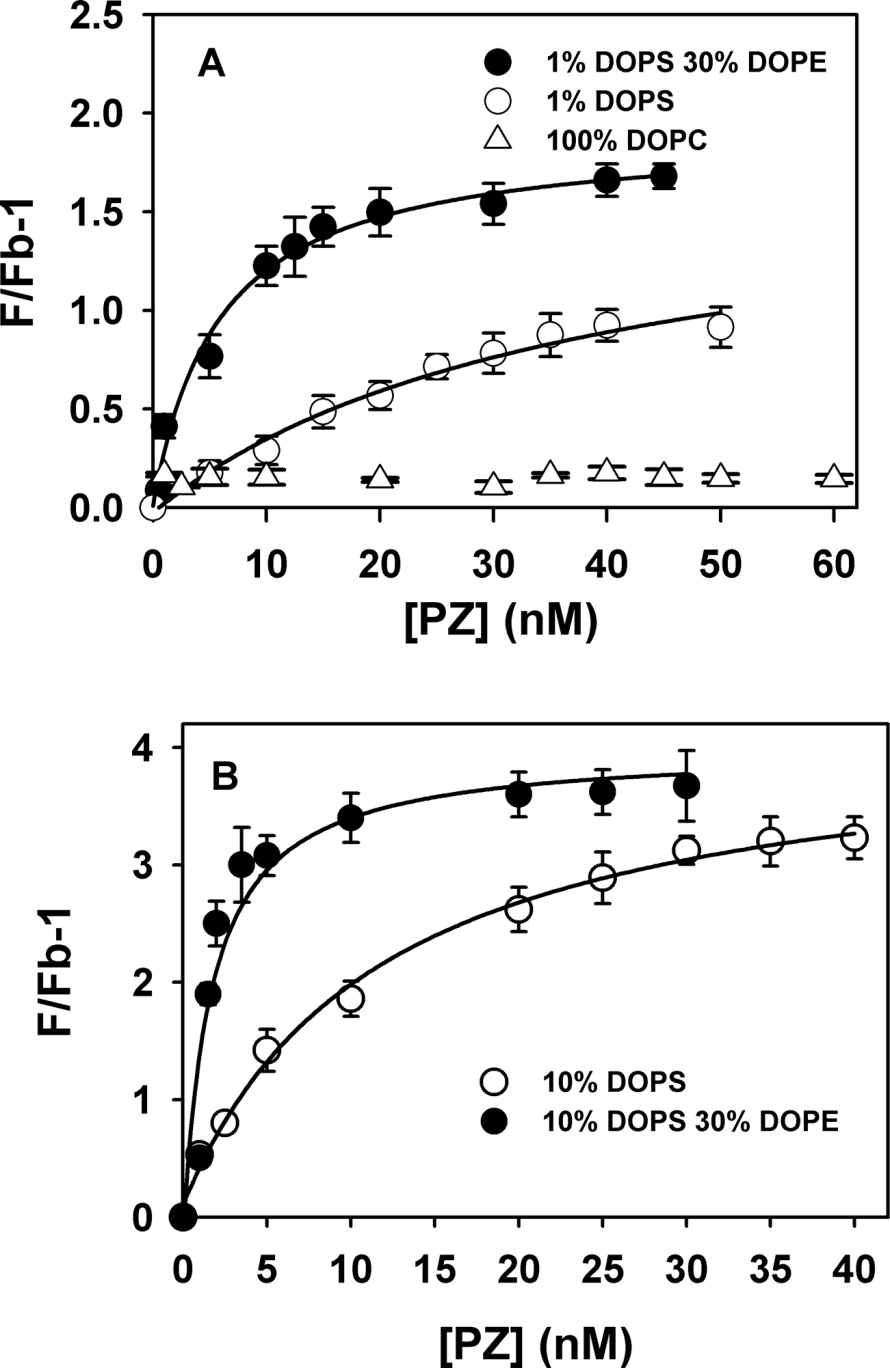
Binding of human PZ to phospholipid vesicles. Fluorescence measurements were performed in 50 mM Tris-HCl, pH 7.5, 175 mM NaCl, 5 mM CaCl_2_, 0.6% PEG by adding increasing concentrations of PZ to 1 μM-labeled DOPC: DOPS: DOPE: Dansyl-PE vesicles of varying composition: A. 69:1:27.5:2.5 (●) 96.5:1: 0: 2.5 (○), 97.5: 0:0:2.5 (Δ); B. 60:10: 27.5: 2.5 (●) and 87.5:10: 0: 2.5 (○).Details of the experimental procedure are described in Methods.

PZ showed a strong affinity towards PS containing vesicles as is evident from the binding *K*_d_'s tabulated in the Table 3. It also bound fairly well to membranes made of DOPE (10–30%) and DOPC (90–70%) in the absence of PS, (binding *K*_d_ for 80: 20 PC: PE and 70: 30 PC: PE are 18 nM and 10 nM respectively). However, the affinity of PZ towards PE:PC membrane is somewhat weaker than PS:PC membrane of comparable PC composition. Binding to PE: PC membranes became tighter with increase in the mol % of PE in the membrane (Table 3 and Fig 3). Interestingly, incorporation of as little as 1 mol % PS in the PC: PE membrane significantly enhanced the affinity of PZ to the membrane. The binding *K*_d_ of PZ to the membrane composed of 60: 30: 10 DOPC: DOPE: DOPS was 1.7 nM, which is comparable to that obtained for the binding to membrane containing 75: 25 DOPC: DOPS (*K*_d_~ 1.0 nM). We were unable to detect any binding of PZ to 100% PC membranes at protein concentrations upto 60 nM (Fig 3A). However, PZ was shown to bind to the membrane by inclusion of 1% PS in the membrane (*K*_d_~ 36 nM. Our membrane binding data thus corroborated nicely with soluble lipid binding data. It was evident from both type of experiments that 1) PZ has stronger affinity towards both PS and PE compared to other membrane phospholipids; 2) PS and PE act synergistically to promote PZ binding to membrane.

**Table 3:** Contribution of PS and PE toward binding of PZ to membrane.*

Membrane	*K*_d_ (nM)
DOPC:DOPE	
90:10	58 ± 5
80:20	18 ± 2
70:30	10 ± 1
DOPC:DOPS	
99:1	36± 7
90:10	17.0 ± 1.4
80:20	1.8 ± 0.1
75:25	1.2 ± 0.2
DOPC:DOPE:DOPS	
69:30:1	6.1 ± 0.8
60:30:10	1.7 ± 0.3

*Binding parameters of PZ to dansyl-PE (2.5%) labeled phospholipid vesicles of varying composition were analyzed as described in Methods. Each data value is an average of three independent measurements.

#### Effect of PS and PE on the interaction between fXa-PZ

**Fig 4:**
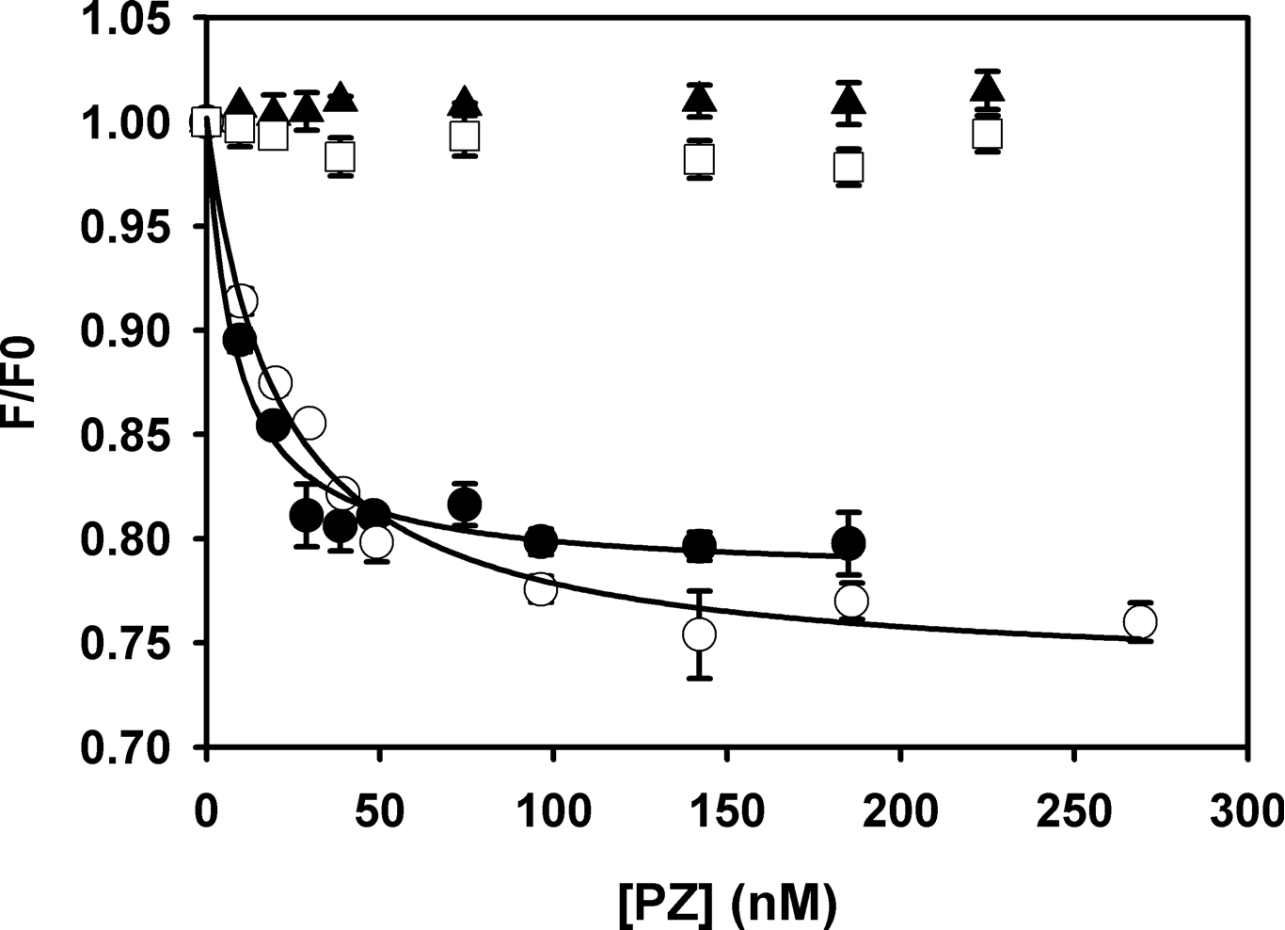
Binding of PZ to DEGR-Xa [(5-(Dimethylamino)-1-naphthalenesulfonyl]-Glutamylycylarginyl)-Xa] on phospholipid membranes. DEGR-Xa (30 nM) fluorescence emission intensities were measured with increasing concentrations of PZ in the presence of 50 μM membrane composed of DOPS: DOPC 30:70 (●), DOPE: DOPC 30:70 (○), 100% DOPC (▼) and in the absence of any membrane (□). Dissociation constants in the presence of PC: PS and PC: PE were 8.6 ± 2.2 nM and 20.6 ± 3 nM, respectively. There was no association of fXa and PZ observed in presence of 100% PC or in absence of any membrane.

For its cofactor activity, it is evident that PZ should bind to phospholipid membranes and form a membrane-bound, stable fXa-PZ complex. Since PS and PE influence membrane binding of PZ, we were interested to test the effect of both lipids on the assembly and formation of the fXa-PZ complex. Binding of PZ to DEGR-Xa on membranes composed of 30:70 PS: PC and 30:70 PE: PC was measured by recording DEGR-Xa fluorescence (Fig 4). The titration curves were fitted to a single-site binding model yielding *K*_d_ values of 8.6±2.2 nM and 20.6 ± 3 nM for PS: PC and PE: PC membranes, respectively. Although both lipids promoted PZ-fXa complex formation, the *K*_d_ for PE was a little higher than that of PS, suggesting that PS is more effective in promoting fXa-PZ complex formation. Such an interaction between fXa and PZ was not observed in the absence of any lipid or in the presence of 100% PC membranes indicating that either PS or PE is specifically needed for the formation of fXa-PZ complex.

#### Effect of PZ on antithrombin (AT) inhibition of fXa in presence of PS and PE

So far we have discussed the efficacy of PS and PE membranes to bind PZ. We have also demonstrated that both PS and PE membranes promote the formation of PZ-fXa complex which is a prerequisite for the cofactor activity of PZ. Here we report a functional assay to provide further evidence in support of the fact that membranes with PS or PE are equally effective in promoting the PZ-fXa interaction.

**Fig 5:**
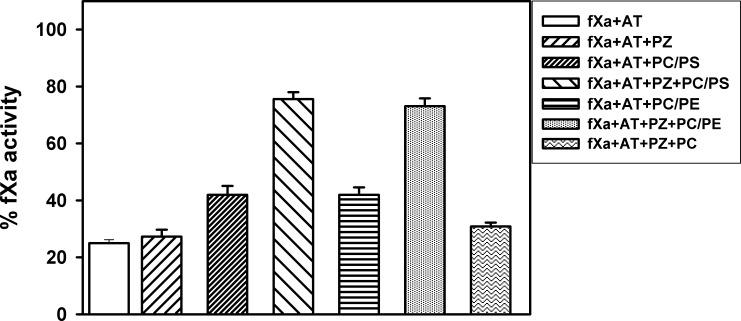
FXa-PZ interaction on phospholipid membranes as measured by AT-mediated inhibition of fXa activity. The amidolytic activity of 5 nM fXa in a buffer containing 50 mM Tris-HCl, pH 7.5, 175 mM NaCl, 5 mM Ca^2+^ and 0.6% PEG was measured in the presence of AT, with or without PZ in the absence and presence of membranes (DOPC: DOPS 70: 30; DOPC: DOPE 70: 30, 100% DOPC) using substrate S2765.

It has previously been reported that, PZ protects fXa from AT-mediated inhibition in presence of Ca^2+^and lipid membrane. This is attributed to fXa binding with PZ on the membrane surface, thereby making fXa less available for AT inhibition [1], [34]. This assay is an alternative means to show the formation of a stable fXa-PZ complex on membranes. Soluble lipids are effective alternatives to membranes for measuring the activities of coagulation proteins. However, inconsistent results were obtained when C6PS and C6PEwere used to perform the AT inhibition assay of fXa in the presence of PZ. Binding of fXa and PZ to the same phospholipid surface is essential for PZ function, but soluble PS or PE probably could not offer an adequate surface to accommodate both proteins. Since PZ can efficiently bind to PE (either soluble or in a membrane), we investigated the efficacy of PZ in restoring AT-mediated fXa inhibition in presence of PE, PS containing membranes, namely, DOPC: DOPE (70: 30) and DOPC: DOPS (70: 30). Fig 5 shows the % inhibition of fXa activity in the presence of PZ, AT and phospholipid membranes of different compositions. We observed a 75% inhibition of fXa activity by AT. Inclusion of PZ produced essentially no effect. In the presence of AT and either of the membranes (PC: PS or PC: PE), there was modest reduction in fXa inhibition. For example, we observed ~40% fXa activity. However, in the presence of both PC: PS and PC: PE membranes, PZ bound to fXa, thus protecting fXa from AT inhibition. The inhibition of fXa activity was 24% in the presence of PS and 27% in the presence of PE. 100% PC membranes, however, were unable to affect antithrombin inhibition of factor Xa in the presence of PZ. Thus it is reconfirmed that either PS or PE should present in the membrane for efficient cofactor function of PZ.

## Discussion

Blood coagulation involves a series of enzymatic reaction taking place on the surface of phospholipid membranes. Formation and activity of enzyme-cofactor complexes such as fIXa:fVIIIa, fXa:fVa, and fVIIa:TF are critically dependent on the availability of relevant phospholipid binding sites on activated platelet membrane and on the lipid composition of the membrane [35–37]. PZ binding to the membrane may also be partly rate determining along with the formation of a membrane-bound fXa-PZ complex for formation of the final ZPI-PZ-fXa ternary complex [19]. Thus, assembly of both PZ and fXa on phospholipid membrane is essential for the effective ZPI inhibition of fXa [8]. The present study systematically analyzes the interaction of PZ with different phospholipids in both membrane and solution phases and provides important information regarding lipid regulation of PZ.

It is well accepted that PS-containing membranes are most suitable for binding the pro- and anti-coagulant proteins; however, PE, PA and phosphorylated derivative of PIP could also serve as important constituents of membranes for some coagulation cascade proteins. Human fVIIa and APC showed significant preference for interacting with PA compared with PS. Sulfogalactosyl sphingosine, a sphingolipid, acts as an anticoagulant lipid that is capable of inhibiting fXa when the enzyme is bound to either fVa or phospholipids [38]. Although the effects of lipids on coagulation proteins and clotting reactions are well recognized, little is known about the effects of those lipids on PZ. Therefore, we aimed to investigate the interaction of different membrane phospholipids, *e*.*g*., PS, PE, PA, PG and PC, with human PZ. PZ bound to PS and PE with similar affinities. Soluble PS and PE (C6PS and C6PE) were used to measure the *K*_d_ values. The *K*_d_ values for binding of fXa to C6PS and C6PE were reported to be 86 μM and 91 μM, respectively, *i*.*e*., the affinities are essentially equal for fXa [16]. Another serine protease, fVIIa, also showed similar behaviors toward C6PS and C6PE (unpublished data). Unlike fXa and fVIIa, fIXa binds to C6PS with 5 times higher affinity compared to C6PE [14]. FVa, the cofactor of fXa, also showed differential binding behavior toward C6PS and C6PE. Binding of soluble lipids to PZ was found to be Ca^2+^ dependent just like other coagulation factors (14,27). We have also checked the binding of other soluble lipids (C6PC, C6PG and C6PA) with PZ. As mentioned in the Results section, PZ bound to those lipids with weaker affinities compared with PS and PE. Also, like other coagulation proteins, PZ binds specifically to L-serine form of PS.

Previous studies have shown that PE enhanced the PS dependence of fX activation by the fVIIa-sTF complex and also promoted fXa binding to membrane. PE, PA, PI, PG were all reported to synergize well with PS in fVIIa-TF activation of fX. Based on that the ABC (Anything But Choline) hypothesis of phospholipid synergy was proposed that predicts that phospholipids with any head group other than choline can synergize with PS [33]. We tested this hypothesis by measuring the binding of PZ to different soluble lipids in combination with a saturating level of C6PS. Our results demonstrated that the binding of PZ to PS and PE is synergistic, *i*.*e*., binding of C6PS to PZ increased the affinity of PZ for C6PE and vice versa. Our findings also suggest that PS and PE binding sites on PZ are not equivalent but are distinct. However, contrary to the ABC hypothesis, binding of PS did not enhance the affinity of PZ for other phospholipids like C6PC, C6PA or C6PG.

Our soluble lipid binding data were further corroborated using a well characterized FRET study to measure binding of PZ to phospholipid vesicles of varying DOPC, DOPS and DOPE composition. Membranes composed of DOPE: DOPC 30:70 supported PZ binding well (*K*_d_~ 10 nM) and incorporation of PS as low as 1% increased the affinity of PZ towards membrane even more (*K*_d_ ~ 6 nM). Thus PE promoted the binding of PZ to the membrane independently of, and cooperatively with, PS. Similarly, we examined whether PE-containing membranes lacking PS can promote the assembly of fXa and PZ. Binding of PZ to DEGR-fXa was followed in the presence of both PE: PC and PS: PC vesicles; *K*_d_ values obtained for fXa-PZ interaction on both type of vesicles suggested that PS and PE individually can support fXa-PZ complex formation. We were unable to detect PZ binding to 100% PC membrane under our experimental conditions. Membranes composed of 100% PC was also unable to assemble fXa-PZ complex on the membrane surface. The hypothesis that both PS and PE can promote fXa-PZ assembly on membranes was again tested using an AT-mediated inhibition assay of fXa in presence of PZ and different phospholipid vesicles. On the lipid surface, PZ forms a complex with fXa that protects fXa from AT inhibition. The protective effect of PZ was used as a measure of fXa-PZ interaction. PZ protected fXa from AT-mediated inhibition in the presence of both membranes; however, the extent of inhibition was less in the presence of PC: PS membrane. Thus, we reconfirmed that both PS and PE could support the formation of a high affinity complex between fXa and PZ.

**Fig 6:**
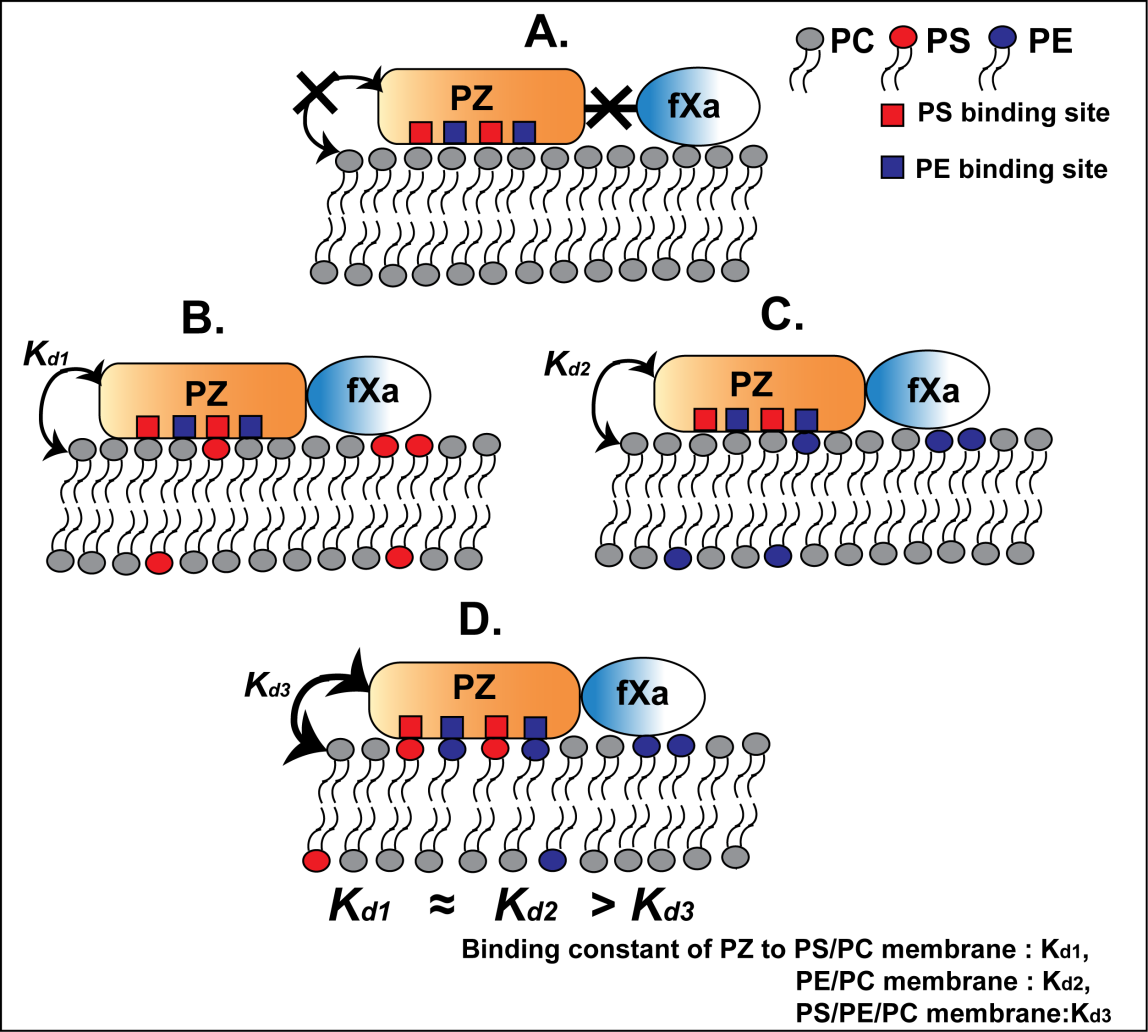
Cartoon diagram describing the major findings of the study. The diagram shows that PZ does not bind to 100% PC membrane and does not form a complex with fXa (A); PZ binds to PS or PE containing membranes with comparable affinity and form a stable PZ-fXa complex (B & C); PS and PE synergistically act to enhance the binding affinity of PZ towards membrane (D).

The impetus for the current study was to provide the groundwork necessary to understand the interaction of PZ with phospholipids, an interaction that is vital to PZ cofactor activity. The current study reveals significant new observations: 1. PZ bound to PS and PE with similar affinities and the binding was observed to be synergistic; 2. Membrane binding of PZ requires the presence of either PS or PE and finally; 3. both PS and PE can support the formation of stable PZ- fXa complex on membrane surface which is absolutely critical for the cofactor activity of PZ. The key messages of the study are also described in Fig 6.

Our finding is particularly interesting considering the trafficking of amino-phospholipids across plasma membrane. The prominent amino-phospholipids of platelet, PS (10%) and PE (28%), are distributed asymmetrically facing the cytosol. On activation of platelet by thrombin during vascular injury these phospholipids are externalized generating a pro-coagulant surface. Lipidomics studies have shown that human platelet, activated by thrombin, collagen or ionophore, externalizes two distinct PS's and five PE's making both PS and PE molecular species abundant on the surface of activated platelets [39]. Thus, it is expected that, PZ will bind *in vivo* with special affinity to activated platelets, due to its cooperative interaction with the lipids. This is an interesting new finding that signifies the role of PZ as an efficient anticoagulant. In future, it will be interesting to study the mechanism of PS/PE synergy with respect to PZ-mediated inhibition of fXa by ZPI in detail which in turn can offer new insights on the physiological role of PZ/ZPI.
